# Genetic Variation among African Swine Fever Genotype II Viruses, Eastern and Central Europe

**DOI:** 10.3201/eid2009.140554

**Published:** 2014-09

**Authors:** Carmina Gallardo, Jovita Fernández-Pinero, Virginia Pelayo, Ismail Gazaev, Iwona Markowska-Daniel, Gediminas Pridotkas, Raquel Nieto, Paloma Fernández-Pacheco, Svetlana Bokhan, Oleg Nevolko, Zhanna Drozhzhe, Covadonga Pérez, Alejandro Soler, Denis Kolvasov, Marisa Arias

**Affiliations:** Centro de Investigación en Sanidad Animal (CISA-INIA), Madrid, Spain (C. Gallardo, J. Fernández-Pinero, V. Pelayo, R. Nieto, P. Fernández-Pacheco, C. Pérez, A. Soler, M. Arias);; National Institute of Veterinary Virology and Microbiology, Pokrov, Russia (I. Gazaev, D. Kolvasov);; National Veterinary Research Institute, Pulawy, Poland (I. Markowska-Daniel);; National Food and Veterinary Risk Assessment Institute, Vilnius, Lithuania (G. Pridotkas);; Belarusian State Veterinary Centre, Minsk, Belarus (S. Bokhan);; State Research Institute of Laboratory Diagnostic and Veterinary Sanitary Expertise, Kiev, Ukraine (O. Nevolko, Z. Drozhzhe)

**Keywords:** African swine fever, genotype II, intergenic regions, genetic variability

## Abstract

African swine fever virus (ASFV) was first reported in eastern Europe/Eurasia in 2007. Continued spread of ASFV has placed central European countries at risk, and in 2014, ASFV was detected in Lithuania and Poland. Sequencing showed the isolates are identical to a 2013 ASFV from Belarus but differ from ASFV isolated in Georgia in 2007.

African swine fever (ASF) is a devastating disease of domestic and wild suids, and there is no vaccine to protect against the disease. ASF is caused by a DNA arbovirus, African swine fever virus (ASFV), belonging to the family *Asfaviridae* (*1*); the virus genome is 170–192 kb long. ASF is endemic in sub-Saharan countries and in Sardinia (Italy) and has become more prevalent in Russia and the Caucasus region (*2*) since its spread from eastern Africa to Georgia (in the Caucasus region) in 2007 (*3*). The ongoing spread of ASFV to adjacent eastern European countries, such as Ukraine (*4*,*5*) and Belarus (*6*), and the uncontrolled spread of the disease in Russia have placed the bordering areas of the European Union at high risk for the introduction of ASFV. In early 2014, the first cases of ASF in the European Union were reported; the cases occurred in 4 wild boars in areas of Lithuania and Poland that border the eastern European country of Belarus (*7*,*8*) (Figure 1). To further our knowledge of the epidemiology and spread of ASFV, we determined the virus sequences of the ASFVs isolated in Poland and Lithuania by using international standardized procedures (*9*) and by the analysis of an additional ASFV genome marker region characterized by the presence of tandem repeat sequences (TRSs). We report the genetic characterization of these ASFVs.

**Figure 1:**
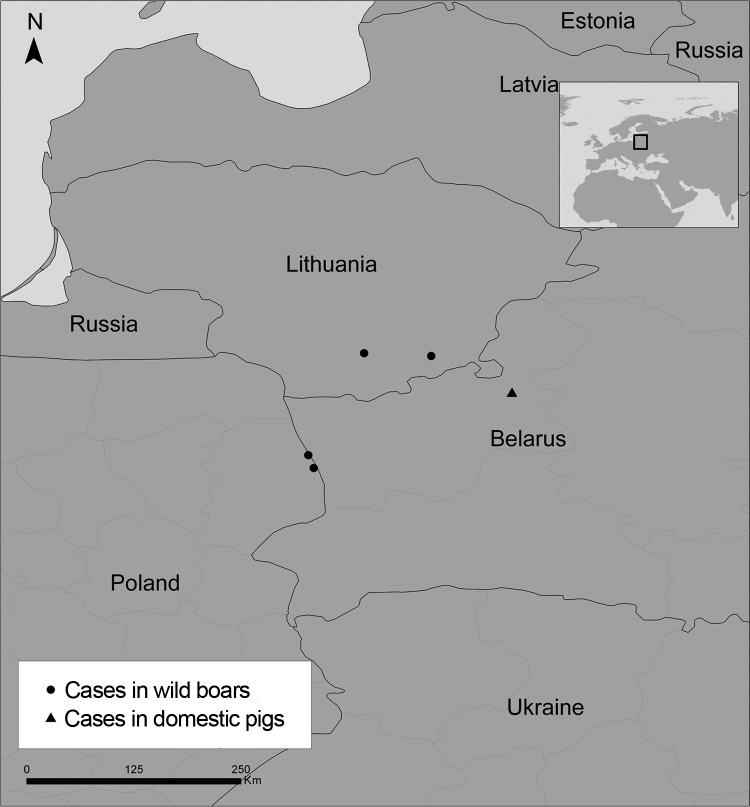
Locations of 4 cases of African swine fever in wild boars in the European Union countries of Poland and Lithuania and location of a 2013 outbreak among domestic pigs in Belarus, an eastern European country that shares a border with Poland and Lithuania. Inset map shows location (square) of countries in the larger map within the larger surrounding area.

## The Study

On January 24, 2014, the European Commission and the World Organisation for Animal Health received reports from Lithuanian authorities of 2 cases of ASF in wild boars. One of the infected animals was found in Salcininkai and the other in Varena, 5 km and 40 km, respectively, from the Belarus border (*7*). Then, on February 14 and 17, 2014, reports of 2 cases of ASF in wild boars were received from northeastern Poland (Sokolka County, Podlaskie Province). One of the infected animals in Poland was found in the municipality of Szudzialowo; the other was found in Kruszyniany, a forest area (*8*). The 2 wild boars in Poland were found dead ≈900 m and ≈200 m, respectively, from Poland’s border with Belarus.

ASFV-positive clinical samples (spleen, kidney, lung, bone marrow) from the 4 infected wild boars were sent to the European Union reference laboratory for ASF, Centro de Investigación en Sanidad Animal (CISA-INIA), Madrid, Spain, for confirmatory testing and genetic characterization. After the presence of ASFV was confirmed in samples, initial genetic characterization was performed by using standardized genotyping procedures on virus DNA extracted directly from homogenized tissues and from bone marrow samples. These analyses included the C-terminal end of the *p72* gene, the full sequence of the *p54* gene, and the central variable region within the *B602L* gene (*9*). We also included in the study 21 genotype II ASFVs that were isolated from wild and domestic pigs in Russia and the Caucasus region during April 2007–June 2013 (Table).

**Table:** African swine fever virus isolates from eastern Europe selected for a study of the genetic variation among genotype II viruses in eastern and central Europe, 2007–2014*

Isolate	Source country, area	Host	Onset of outbreak	GenBank accession no.		
				p72 gene	P54 gene	CVR
Abk07	Georgia, Abkhazia Republic, Gulripish	DP	2007 Jul 04	JX857509	JX857495	JX857523
Arm07	Armenia, Dilijan	DP	2007 Aug 07	JX857508	JX857494	JX857522
Che07	Russia, Chechnya Republic, Shatoysky	EWB	2007 Dec 04	JX857510	JX857496	JX857524
Az08D	Azerbaijan, Qebele District	DP	2008 Jan 22	JX857515	JX857501	JX857529
Az08B	Azerbaijan, Qebele District	DP	2008 Jan 22	JX857516	JX857502	JX857530
Ing08	Russia, Ingushetia Republic, Sunzhensky	EWB	2008 Jul 21	JX857511	JX857497	JX857525
Oren08	Russia, Orenburg Oblast, Chernorechye	DP	2008 Jul 10	JX857512	JX857498	JX857526
NO08/Av	Russia, Republic of North Osetia, Vladikawkaz	DP	2008 Jul 18	JX857513	JX857499	JX857527
NO08/Ap	Russia, Republic of North Osetia, Prigorodni	DP	2008 Jul 21	JX857514	JX857500	JX857528
Dagestan09	Russia, Dagestan Republic, Tarumovsky, District	EWB	2009 Sep 11	JX857517	JX857503	JX857531
StPet09	Russia, Leningradskaya Oblast, Kirovsky	DP	2009 Oct 01	JX857520	JX857506	JX857534
Kalmykia09	Russia, Republic of Kalmykia, Yashaltinsky district	DP	2009 Oct 10	JX857519	JX857505	JX857533
Rostov09	Russia, Rostov Oblast, Krasnosulinsky District	DP	2009 Oct 20	JX857518	JX857504	JX857532
Tver0511/Torjo	Russia, Tver Oblast, Torjo	DP	2011 May 31	KJ627208	KJ627186	KJ627197
Tver0312/Novo	Russia, Novozavidovskii, Tver region	DP	2012 Mar 14	KJ627212	KJ627190	KJ627201
Tver0312/Torjo	Russia, Torjo, Tver region	EWB	2012 Mar 28	KJ627211	KJ627189	KJ627200
Tver0712/Les	Russia, Lesnoi, Tver region	DP	2012 Jul 16	KJ627210	KJ627188	KJ627199
Ukr12/Zapo	Ukraine, Zaporozhye region	DP	2012 Jul 30	JX857521	JX857507	JX857535
Tver0812/Bolo	Russia, Bologovskii, Tver region	EWB	2012 Aug 15	KJ627209	KJ627187	KJ627198
Tver1112/Zavi	Russia, Zavidovo, Tver region	EWB	2012 Nov 20	KJ627214	KJ627191	KJ627202
Bel13/Grodno	Belarus, Grodno region, Lelyukinskiy District of Ivye	DP	2013 Jun 19	KJ627215	KJ627192	KJ627203
LT14/1490	Lithuania, Šalčininkai District Municipality	EWB	2014 Jan 21	KJ627216	KJ627193	KJ627204
LT14/1482	Lithuania, Alytus County, Varėna District Municipality	EWB	2014 Jan 21	KJ627217	KJ627194	KJ627205
Pol14/Sz	Poland, Szudzialowo, Sokolka County, Podlaskie Province	EWB	2014 Feb 14	KJ627218	KJ627195	KJ627206
Pol14/Krus	Poland, Kruszyniany, Sokolka County, Podlaskie Province	EWB	2014 Feb 17	KJ627219	KJ627196	KJ627207

*CRV, central variable region; DP, domestic pig; EWB, European wild boars.

**Figure 2:**
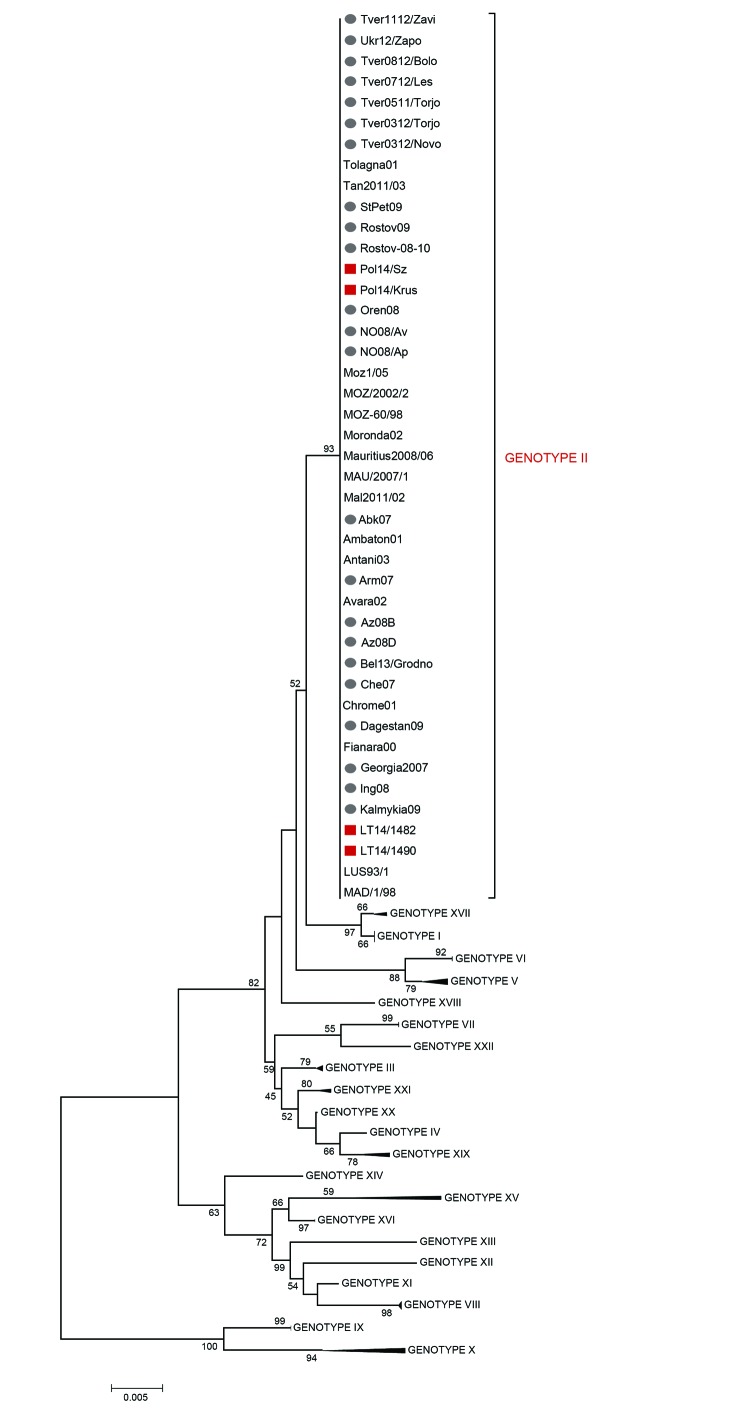
Minimum evolution (ME) phylogenetic tree of African swine fever virus (ASFV) isolates from Lithuania and Poland based on the C-terminal end of the *p72* coding gene relative to the 22 p72 genotypes (labeled I-XXII), including 88 nt sequences. The tree was inferred by using the ME method (http://www.megasoftware.net/mega4/WebHelp/part_iv___evolutionary_analysis/constructing_phylogenetic_trees/minimum_evolution_method/rh_minimum_evolution.htm) following initial application of a neighbor-joining algorithm. The phylogenetic tree was rooted by the midpoint method. The percentage of replicate trees >50% in which the associated taxa clustered together by bootstrap analysis (1,000 replicates) is shown adjacent to the nodes. The robustness of the ME tree was tested by using the close-neighbor-interchange algorithm at a search level of 1. Squares indicate ASFV isolates from Lithuania and Poland that were genotyped in this study; circles indicate ASFV isolates during 2007–2013 from the Caucasus region. Scale bar indicates nucleotide mutations per site.

We compared the nucleotide sequences obtained from the p72- and p54-based PCRs with those of previously described representative isolates (*10*). We used Clustal Omega (http://www.clustal.org/) to perform multiple sequence alignments. Minimum evolution trees, rooted at the midpoint, were constructed by using MEGA V6.0 (http://www.megasoftware.net/) with the *p*-distance nucleotide substitution model. The 2014 ASFVs from Lithuania (LT14/1482, LT14/1490) and Poland (Pol14/Sz and Pol14/Krus) clustered, as expected, within p72 genotype II (Figure 2) and showed 100% nucleotide identity with all compared ASFV isolates from eastern Europe across the 478-bp C-terminal *p72* gene and the 558-bp full length *p54* gene. We obtained the same result by sequencing the central variable region within the *B602L* gene, revealing 10 copies of amino acid tetramer repeats that were 100% identical and unique to those of the ASFV circulating in the Caucasus regions since 2007 (*11*).

**Figure 3:**
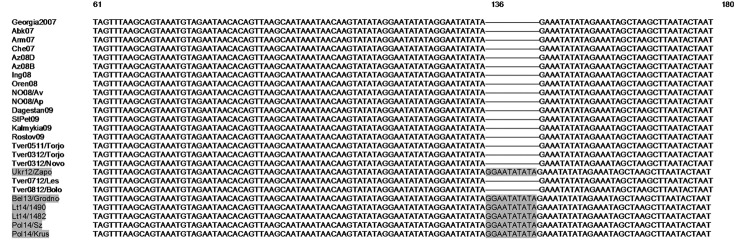
Partial nucleotide sequence alignment of the intergenic region between *I73R* and *I329L* in African swine fever virus (ASFV) isolates from eastern and central Europe, including a virus isolated in 2007 in Georgia (Georgia2007; GenBank accession no. FR682468.1). The mutation that results in the insertion of a single nucleotide internal repeat sequence (GGAATATATA) in the ASFVs from Belarus, Ukraine, Lithuania, and Poland is indicated by gray shading.

Although the central variable region has proven useful for resolving epidemiologic complexities at the genotype (*12*), country (*13*), and region levels, additional genome markers are required to determine the origin and to map the spread of closely related ASFV isolates circulating in eastern Europe. Thus, we designed a set of primers, named ECO1A (5′-CCATTTATCCCCCGCTTTGG-3′ binding site 172,270–172,290) and ECO1B (5′-TCGTCATCCTGAGACAGCAG-3′ binding site 172,616–172,626), to amplify a 356-bp fragment located between the *I73R* and *I329L* genes and characterized by the presence of TRS (*14*). Primer binding sites were based on the genome of the ASFV from Georgia (GenBank accession no. FR682468.1). Using the same reaction conditions as used for full *p54* gene amplification (*10*) and an annealing temperature of 60°C, we generated 367-bp amplicons from isolates from Ukraine, Belarus, Lithuania, and Poland. The estimated size of the remaining isolates from eastern Europe that were included in the study was 356 bp (data not shown). Nucleotide sequence analysis of the PCR products revealed that the size difference was caused by the insertion of an additional TRS (GGAATATATA) at nt 136 (Figure 3). All sequences generated in this study were submitted to GenBank under accession nos. KJ620028–51.

## Conclusions

Current available molecular data derived by using standardized genotyping procedures (*9*) have indicated the presence of only 1 ASFV variant. That variant belongs to p72 genotype II, which has been circulating in eastern European countries since the introduction of ASFV into Georgia in 2007 (*11*). In agreement with those findings, results from our analysis of the 3 independent regions included in the classical genotyping showed that sequences for ASFV isolates from Lithuania and Poland were 100% homologous with those for ASFVs from eastern Europe. However, the long-term presence of ASFV in Russia and the Caucasus regions and the rapid spread of the virus to neighboring countries highlight the need for finding additional ASFV genome markers capable of discriminating among circulating virus isolates so that we may better determine their source and evolution. 

The whole-genome sequence analysis of ASFV has identified some regions that contain tandem repeat arrays that have proven useful for discriminating between closely related ASFVs (*15*). Thus, the approach described in our study focused on analysis of the TRS in the intergenic region between the *I73R* and *I329L* genes at the right end of the genome (*14*). The results showed that the viruses from Poland and Lithuania had a TRS insertion identical to that present in ASFV isolates from Belarus and Ukraine. This TRS insertion was absent in the remaining viruses from eastern Europe, including those obtained in Tver Oblast, Russia, in 2012 and in Georgia in 2007. These molecular data, together with the epidemiologic findings, confirmed that the ASFVs detected in Poland and Lithuania most probably originated from Belarus. However, knowledge of the epidemiology of ASF and a full understanding of the evolution and spread of ASFV in this region require additional sequence analysis of ASFVs currently circulating in Russian regions bordering Belarus and Ukraine.

Our results show the genetic variability among ASFVs circulating in eastern Europe and describe a new method that can be useful for distinguishing between closely related ASFV isolates. Such genetic data are essential for determining the source and studying the evolution of ASFV isolates and to fully elucidate the spread of ASFV in the eastern and central European countries.
